# Methods in molecular biology: minor errors in primer citations with major consequences: how can we minimize these mistakes?

**DOI:** 10.1054/bjoc.1999.1186

**Published:** 2000-04-03

**Authors:** M Jung, J M Muche, K Jung, S A Loening

**Affiliations:** Departments of Urology, Dermatology, University Hospital Charité, Humboldt University Berlin, Schumannstr. 20/21, Berlin, D-10098, Germany


DOI: 10.1054/ bjoc.1999.1185, available online at http://www.idealibrary.com on IDEAL®

### Induction of T-cell mitogenic unresponsiveness by recombinant human granulocyte colony-stimulating factor – a reply

**Sir**,

We are grateful that Rutella et al have brought their impressive research to our attention. We regret that we were not aware of their work prior to the submission of our other recent publication (Reyes E et al, 1999). As they note in their letter to the Editor, combining our investigative results suggests that rHuG-CSF exerts, through the induction of soluble factors such as IL-1 receptor antagonist and TNF soluble receptor, a reversible inhibitor effect on PBMC proliferation that is not due to alterations in cell number ($CD3^+$, $CD19^+$, $CD45^+$, $CD14^+$), cytokine production (IL-1, IL-2, IL-6, IL-10, TNF-α, or IFNγ), or activation status (HLA-DR, CD57, or IL-2 receptor α expression). Rutella et al suggest that the decreased PBMC proliferative response to mitogen after treatment with sera from patients treated with rHuG-CSF is due to cell cycle arrest such that a lymphocyte partial activation phenotype becomes dominant. Interestingly, while failure to progress through $G_0$ is commonly due to alterations in IL-2R expression, we found that rHuG-CSF treatment does not alter the expression of IL-2 receptor alpha (IL-2Rα) on PBMCs. Certainly other avenues leading to inhibition of lymphocyte cycling will have to be explored. Our finding of an upregulation of memory ($CD45RO^+$) T helper cells ($CD4^+$) with rHuG-CSF treatment with a concomittant decline in naive ($CD4^+CD45RA^+$) subset may explain the decreased proliferative response to mitogens, since naive cells demonstrate greater proliferation to mitogenic stimulation than do memory cells. However, this finding cannot explain the lymphocyte cycling arrest in $G_0$. Evaluation of the effects of rHuG-CSF treatment on the expression of co-regulatory molecules on antigen presenting cells may prove fruitful in elucidating the mechanism of lymphocyte cycling arrest.

The ability of rHuG-CSF to inhibit mitogenic proliferative responses may be proven useful clinically in inhibiting unwanted immunologic activity such as acute graft-versus-host disease, autoimmune disorders, as well as chronic inflammatory diseases. Further, Rutella et al's suggestion that rHuG-CSF results in tolerance induction may be exploited by coupling rHuG-CSF treatment with specific antigens in autoimmune disorders. Certainly, more work is needed to establish the mechanism of rHuG-CSF's effect on mitogenic proliferative responses, as well as testing the effect in the context of specific antigens. Still, the previous work of investigators such as Rutella et al, Pan et al, and Roe et al, as well as our own, demonstrates a fruitful role for rHuG-CSF as an immunomodulator in the context of autoimmune/allergy and transplant medicine.

*E Reyes, ED Bernstein, M Alvarez-Mon*
*Medicine/Immune System Diseases Oncology Service,*
*Department of Medicine,*
*'Principe de Asturias' University Hospital,*
*Alcalá University, 28871 Alcalá de Henares, Madrid, Spain*

### REFERENCES

Reyes E, Garcia-Castro I, Esquivel F, Hornedo J, Cortes-Funes H, Solovera J and Alvarez-Mon M (1999) Granulocyte colony-stimulating factor (G-CSF) transiently suppresses mitogen-stimulated T-cell proliferative response. *Br J Cancer* **80**(1/2): 229–235

DOI: 10.1054/ bjoc.1999.1186, available online at http://www.idealibrary.com on IDEAL®

### Methods in molecular biology: minor errors in primer citations with major consequences: how can we minimize these mistakes?

**Sir**,

We read with great interest the article of Forsyth et al (1999) (*Br J Cancer* **79**: 1828–1835) about the role of gene expression of matrix metalloproteinases (MMPs) in malignant gliomas. There is a growing interest in detecting gene expression of these components for a better understanding of molecular mechanisms regarding tumour invasion and metastasis in malignant diseases (Parsons et al, 1997). Therefore, sensitive and specific molecular biological methods are required but they are mostly user orientated and hardly standardized.

We are investigating the molecular biology of prostate cancer, in particular MMPs and their tissue inhibitors, by real-time RT-PCR (Wittwer et al, 1997). Applying the primers for MMP-2 (also named gelatinase A or collagenase IV) used by Forsyth et al for this purpose, we experienced some disagreeable surprises which seem important enough to us to be commented upon.

© 2000 Cancer Research Campaign

We were interested in the characteristics of Forsyth's primer pair and checked it by using the software 'Primer 3' as found on the internet (http://www.genome.wi.mit.edu/cgi-bin/primer/primer3_www.cgi). 'Primer 3' picks polymerase chain reaction (PCR) primers amplifying a particular region of a target gene defined from a nucleotide sequence database, generates scores for complementarities of the primer pairs and calculates melting temperatures and GC-contents of primers and products. Important for that is the complete entry of mRNA sequence of the target gene from a molecular biology database (Burks, 1999).

In the case of the MMP-2 primers from Forsyth et al, 'Primer 3' could not identify the binding sites of both primers in the source MMP-2 mRNA sequence (Accession number [AC]: JO3210; identification code [ID]: HUMCN4GEL). Putative binding sites of both MMP-2 primers were therefore checked by a Blast-Internet program (http://www.ncbi.nlm.nih.gov/cgi-bin/BLAST/nph-new-blast) comparing a nucleotide query sequence against a nucleotide sequence database. The applied software falls back on four databases (non-redundant sequences from GenBank, EMBL, DDBJ and PDJ). To our surprise, the used MMP-2 primer sequences of Forsyth et al produced significant alignments with regions of the human tissue inhibitor TIMP-1 gene, exon 1 (AC: L47357; ID: HUMTIMP1G), the human collagenease inhibitor mRNA (AC: M59906; ID: HUMOGCA) and the human fibroblast collagenase inhibitor mRNA (AC: M12670; ID: HUMFCI) but not with the MMP-2 gene. However, the alignments with both collagenase inhibitors were found only in 22 of the 25 input bases of the primers and the Blast-program automatically cut off the first three bases.

After comparing the source mRNA sequence for both collagenase inhibitors mentioned above and the completed sequences of the primers, we found one false base at the third position of the 5′- (A instead of G) and at the 3′-primer (C instead of A) both at the 5′-end. We also observed a print error in the reading direction of the gelatinase A reverse primer (5′ instead of 3′). Consequently, the size of the amplification product would be 686 bp instead of the published product size of 473 bp.

In consequence of our investigation, we are uncertain whether the authors really detected MMP-2 and not TIMP-1. The agarose gel bands shown in Figure 1 cannot be considered as proof for the detection of the target gene and for the correct PCR product size since no DNA molecular weight markers are present in the same gel run.

In addition to these data, we found that the product size for MT1-MMP as cited in the same paper is not 548 bp but 530 bp and that the used 5′-primer for this gene contains a false cited base in the third position from the 3′-end (G instead of C).

Apart from drawing the interested reader's attention to these possible mistakes, the paper gives rise to the following general recommendations:

- The selection of primers has to be documented in an easily understandable manner to avoid serious mistakes
- Therefore, primer sequences should be specified by declaration of the source of the target gene sequence (e.g. the nucleotide sequence database used) and of the primer position within the target sequence
- In addition, citation of the accession number (AC) and the identification code (ID) of the investigated gene sequence would give a definite relation between a nucleotid sequence and its target gene.

Printing errors of nucleotide sequences of primers could thus more easily be checked, and the suggested procedure gives a better guarantee for correct citations and successful application of published methods in molecular biology.

*M Jung[1], JM Muche[2], K Jung[1] and SA Loening[1]*
*Departments of [1]Urology and [2]Dermatology,*
*University Hospital Charité,*
*Humboldt University Berlin, Schumannstr. 20/21,*
*D-10098 Berlin, Germany*

#### REFERENCES

Burks C (1999) Molecular Biology Database List. *Nucleic Acids Res* **27**: 1–9

Forsyth PA, Wong H, Laing TD, Rewcastle NB, Morris DG, Muzik H, Leco KJ, Johnston RN, Brasher PMA, Sutherland G and Edwards DR (1999) Gelatinase-A (MMP-2), gelatinase-B (MMP-9) and membrane type matrix metalloproteinase-1 (MT1-MMP) are involved in different aspects of the pathophysiology of malignant gliomas. *Br J Cancer* **79**: 1828–1835

Parsons SL, Watson SA, Brown PD, Collins HM and Steele RJC (1997) Matrix metalloproteinases. *Br J Surg* **84**: 160–166

Wittwer CT, Ririe KM, Andrew RV, David DA, Gundry RA, and Balis UJ (1997) The LightCycler™: a microvolume multisample fluorimeter with rapid temperature control. *Bio Techniques* **22**: 176–181

DOI: 10.1054/ bjoc.1999.1187, available online at http://www.idealibrary.com on IDEAL®

### Methods in molecular biology: minor errors in primer citations with major consequences: how can we minimize these mistakes? – reply

**Sir**,
I am replying to the above letter, which was sent concerning our paper 'Gelatinase-A (MMP-2), gelatinase-B (MMP-9) and membrane type-matrix metalloproteinase-1 (MT1-MMP) are involved in different aspects of the pathophysiology of malignant gliomas', Forsyth PA et al (1999) *Br J Cancer* **79**: 1828–1835.

Jung et al point out that the primer sequences listed in our paper for reverse transcription polymerase chain reaction (RT-PCR) amplification of human gelatinase-A (MMP-2) are incorrect. The sequences given in fact correspond to primer sequences used for human TIMP-1 amplification, with additional *Bam*HI and *Hin*DIII cloning sites at the 5′-end of the oligonucleotides to allow

 © 2000 Cancer Research Campaign

